# CIEDE2000 colour difference formula for surface colour analysis of low-carbon grey and white portland cement pastes with high-volume GGBS replacement

**DOI:** 10.1038/s41598-026-56426-4

**Published:** 2026-06-11

**Authors:** Payam Shafigh, Poh-Yee Loh, Yong Jin, Soon Poh Yap

**Affiliations:** 1 Department of Civil Engineering, College of Architecture and Energy Engineering, Wenzhou University of Technology, Wenzhou, 325000 Zhejiang China; 2 Centre for Building, Construction & Tropical Architecture (BuCTA), Faculty of Built Environment, Universiti Malaya, Kuala Lumpur, 50603 Malaysia; 3 Department of Civil Engineering, Faculty of Engineering, Universiti Malaya, Kuala Lumpur, 50603 Malaysia

**Keywords:** CIEDE2000, Colour uniformity, Curing, Green construction materials, High-volume GGBS, Engineering, Environmental sciences, Materials science

## Abstract

The global movement towards sustainable concrete is accelerating the adoption of low-carbon cement within the construction sector. Many engineering properties of low-carbon cement-based materials (CBMs) containing supplementary cementitious materials have been extensively studied and are well understood, but information regarding colour properties is limited. In this study, a colour analysis was conducted on cement paste incorporating ground granulated blast furnace slag (GGBS) as replacement material for ordinary Portland cement (OPC) and white Portland cement (WPC). The replacement levels were set at 0%, 30%, 50% and 70% by OPC or WPC volume. Results from CIEDE2000 colour difference formula (∆*E*_00_) showed that the water-cured WPC paste had highest colour uniformity (∆*E*_00_ = 0.10 ± 0.03), whereas the OPC paste had the lowest (∆*E*_00_ = 1.29 ± 0.76). GGBS addition increases the lightness (L*) of the OPC paste but decreases the L* of the WPC paste at similar rates. The highest colour difference (∆*E*_00_) between a control mix (has only OPC or WPC) and mixes containing 70% GGBS was approximately 7. When red iron oxide pigment was added, the ∆*E*_00_ value dropped to approximately 3.7 for the WPC mix and 1.5 for the OPC mix. This result shows that a notable colour change was observed when Portland cement was replaced with high-volume GGBS in the cement pastes, but this colour difference was considerably reduced in pigmented mixtures. This interesting result reveals that in construction products, Portland cement can be replaced with high-volume GGBS without compromising the products’ surface colour.

## Introduction

Cement clinkers are normally produced in either grey or white variants for the manufacture of grey or white Portland cement (WPC). Owing to the higher cost of WPC, it is primarily used in applications where the aesthetic of the final product is important, such as fair-faced concrete (also known as architectural concrete), decorative concrete (e.g. exposed aggregate concrete and stamped concrete), coloured concrete and precast panels. In 2024, WPC accounted for the highest market share (59.5%) in the white cement industry, followed by white masonry cement, white Portland limestone cement and others, such as white calcium aluminate cement^1^. WPC is mostly used because of its high strength and exceptional finish, particularly in terms of colour homogeneity and brightness.

The global movement towards sustainable concrete has prompted numerous studies focusing on the partial replacement of cement with supplementary cementitious materials (SCMs) while reducing reliance on natural resources^2^. Among the various types of SCMs, silica fume, ground granulated blast furnace slag (GGBS), fly ash and metakaolin are widely recognised and used in the construction industry. Hence, numerous studies have investigated the fresh, mechanical, microstructural and durability properties of cement-based materials (CBMs) and structural performance of concrete containing these SCMs. However, studies related to the chromatic properties of all types of CBMs with and without SCMs, especially those blended with WPC, are limited.

Chromatic analysis for CBMs is conducted mainly to determine the influence of raw materials (e.g. binder, aggregate, water, colourant and admixtures) to the appearance of final products^3–4^ and is considered a non-destructive method for assessing the deterioration of CBMs through observed colour changes^5–6^. The colour of an unpigmented CBM is mostly determined by the colour of cement rather than the colour of aggregates^7–8^. However, other factors influence the surface colours of CBMs. Schack and Haist^9^ reported that binder colour generally lightens after hydration. A bright CBM can be produced by using light-coloured fine aggregates^4,8^ or by increasing the water-to-cement (w/c) ratio^7,10^. CBMs with different colours can be produced by adding colourants, such as commercial pigments^11^, special-purpose pigments^12^ and waste materials^13^.

As concrete undergoes ageing or accelerated deterioration, its surface appearance changes. After 10 years of exposure to an industrial environment, the concrete surfaces became 12%–14% darker because of physical degradation on the surface^14^. Additionally, the growth of microorganisms that form black biofilm on the surface^15^ and the formation of efflorescence^16^ after weathering affect the overall appearance of a CBM. Concrete deterioration through fire damage^6^, abrasion^17^ and acid attack^18^ is normally accompanied by a change in appearance. At elevated temperatures, the grey OPC mortar darkened at 300 °C and gradually lightened at 800 °C^6^. In the acid attack test, the colour difference (∆*E*) of the OPC mortar increased with extension of test duration (56 days)^18^. This may be due to the increase in cracks and pores (matrix decomposition) on and near the concrete surface that affect the light reflection, as well as the chemical reactions that alter the colour of concrete^6,18^. Therefore, the possibility of using colour measurement as a predictive tool for assessing the level of deterioration has been explored. However, analytic approaches vary because of differences in colour measurement techniques, choice of colour space and colour attributes. Banerjee et al.^5^ correlated the residual strength of concrete after immersion in various chemicals with specific colour parameters. Meanwhile, Annerel and Taerwe^19^ and Carré et al.^20^ used different colour parameters to map changes in concrete colour at various elevated temperatures.

Previous studies related to CBM colour have focused on the surface colours and strength properties of CBMs after the addition of pigments. However, information on colour changes due to ageing and under different curing conditions is limited, particularly when a type of cementitious materials, such as GGBS, is incorporated into the mixtures. Understanding changes in physical appearance during curing is essential not only for the quality monitoring of exposed concrete but also for determining the suitable time for the colour measurement of a newly cast sample. In addition, most studies on CBMs, such as Akbulut et al.^6^, López et al.^14^ and Tang et al.^21^ adopted ∆*E*_76_ instead of CIEDE2000 (∆*E*_00_) colour difference formula, which has a higher correlation with human visual perception^22–23^. The main aim of this research is to evaluate the suitability of using GGBS as ordinary Portland cement (OPC) or WPC replacement (up to 70%) in CBM production from a chromatic viewpoint. The test specimens were prepared in order to meet the requirements of the testing instruments (spectrophotometer and flatbed scanner) and to eliminate the influence of external factors, such as light leakage during colour measurement, and contamination on the sample surface due to weathering and the application of release agents. The ∆*E*_00_ value was used, which can aid researchers and practitioners in quantitatively assessing the influence of binders on the aesthetic aspects of unpigmented and pigmented CBMs. It can contribute to the development of ∆*E*_00_ scale for colour analysis on CBMs.

### Overview on CIELAB colour space

Colour can be quantified using different colour spaces, such as CIELAB, RGB, HSI and Munsell colour system. Of the colour spaces, only the CIELAB colour space offers an approximately uniform visual spacing and quantifies colour difference (∆*E*) in a way that closely reflects human visual perception^24–25^. Therefore, it is the mostly used colour space for reporting and analysing CBM colour.

**Fig. 1:**
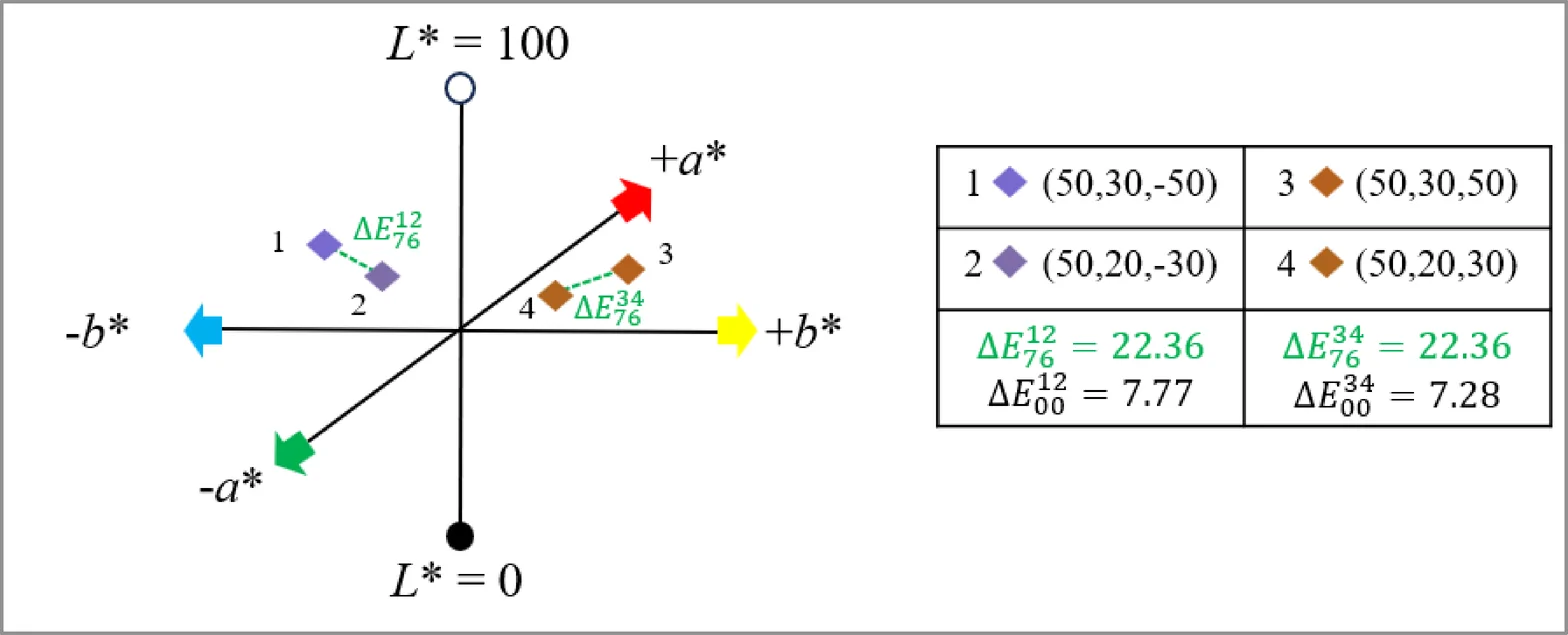
CIELAB colour space and illustration of Euclidean distance (∆*E*_76_) for colour 1, 2, 3 and 4 as example. The colour represented by the four points and ∆*E*_00_ value were determined using www.colormine.org. (source: authors).

The CIELAB colour space was introduced by International Commission on Illumination in 1976. In this system, colour is represented using Cartesian coordinates of lightness–darkness (L*), redness–greenness (a*) and yellowness–blueness (b*)^26^. As illustrated in Fig. 1, lightness (L*) ranges from 0 to 100, and a small value denotes a dark colour. The a* and b* values range from negative values to positive values, and colour saturation increases with absolute value. The axes are designated as follows: redness (+a*), greenness (–a*), yellowness (+b*) and blueness (–b*). Achromatic colours, such as white, black and grey, have small a* and b* values that is close to zero.

The difference between two colours can be calculated using the CIELAB (also known as CIE L*a*b*) value. When CIELAB was first introduced, the colour difference (∆*E*_76_) was calculated on the basis of Euclidean distance, as demonstrated in Eq. (1) and illustrated in Fig. 1. A large ∆*E*_76_ value denotes a considerable difference. The formula for colour difference has been improved several times, and the latest version is ∆*E*_00_, which was introduced in 2000, named CIEDE2000, as shown in Eq. (2)^27^. The new formula was aimed at generating ∆*E* value close to human visual perception^25–26^. As illustrated in Fig. 1, although the Euclidean distance between points 1 and 2 is the same as that between points 3 and 4, the visualised colour difference for the latter is less significant (∆*E*_00_ = 7.28). Given the complexity of Eq. (2), the ∆*E*_00_ was calculated using a Microsoft Excel spreadsheet prepared by Sharma et al.^28^.

1 $$\:\varDelta\:{E}_{76}\left({L}_{1}^{\mathrm{*}},{a}_{1}^{\mathrm{*}},{b}_{1}^{\mathrm{*}};{L}_{2}^{\mathrm{*}},{a}_{2}^{\mathrm{*}},{b}_{2}^{\mathrm{*}}\right)=\varDelta\:{E}_{76}^{12}=\sqrt{{(\varDelta\:{L}^{\mathrm{*}})}^{2}+{(\varDelta\:{a}^{\mathrm{*}})}^{2}+{(\varDelta\:{b}^{\mathrm{*}})}^{2}}$$

2 $$\:\varDelta\:{E}_{00}\left({L}_{1}^{\mathrm{*}},{a}_{1}^{\mathrm{*}},{b}_{1}^{\mathrm{*}};{L}_{2}^{\mathrm{*}},{a}_{2}^{\mathrm{*}},{b}_{2}^{\mathrm{*}}\right)=\varDelta\:{E}_{00}^{12}=\sqrt{{\left(\frac{\varDelta\:{L}^{{\prime\:}}}{{k}_{\mathrm{L}}{S}_{\mathrm{L}}}\right)}^{2}+{\left(\frac{\varDelta\:{C}^{{\prime\:}}}{{k}_{\mathrm{C}}{S}_{\mathrm{C}}}\right)}^{2}+{\left(\frac{\varDelta\:{H}^{{\prime\:}}}{{k}_{\mathrm{H}}{S}_{\mathrm{H}}}\right)}^{2}+{R}_{T}\left(\frac{\varDelta\:{C}^{{\prime\:}}}{{k}_{\mathrm{C}}{S}_{\mathrm{C}}}\right)\left(\frac{\varDelta\:{H}^{{\prime\:}}}{{k}_{\mathrm{H}}{S}_{\mathrm{H}}}\right)}$$

No standardised guideline or customised study about CBM classification based on ∆*E* has been established possibly because of the heterogeneous nature of CBMs and their non-uniform colour and porous or uneven surfaces that easily affect colour measurements. Previous studies considered a ∆*E*_76_ value below 1.5 as insignificant colour difference for concrete^15,29^. However, the corresponding reference value for ∆*E*_00_ has not been established. In general, ∆*E*_00_ is smaller than ∆*E*_76_^30^. Most studies on CBMs have applied ∆*E*_76_ instead of ∆*E*_00_, while studies regarding the correlation between human visual perception on printed samples^22^ and wood samples^23^ demonstrated that ∆*E*_00_ is more accurate than ∆*E*_76_. Therefore, ∆*E*_00_ was adopted in the present study.

### Colour measurement of CBMs through spectrophotometry

Colour is a visual perception influenced by an object, light source and observer. A spectrophotometer measures the spectral reflectance of a confined surface with a known light source. It then calculates the numerical value of a colour for a specific colour space on the basis of specified illuminant and observer angle. Spectral reflectance is a normalised measure showing how a surface is reflective across different wavelengths. It is defined as the ratio of the amount of reflected light by an object to the amount of incident light^31^. The colour of an opaque material is determined by the wavelengths of visible light (approximately 380–780 nm) that it reflects^26^.

**Fig. 2:**
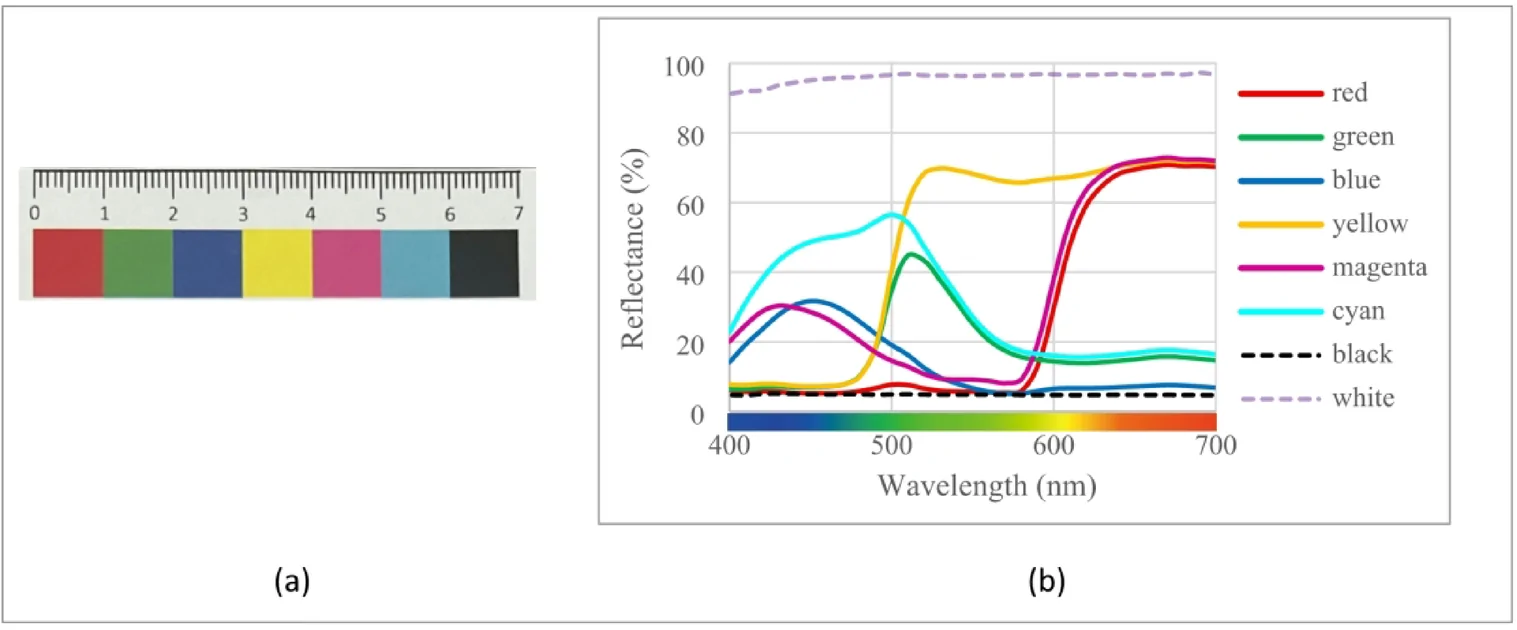
(**a**) Customised colour monitoring ruler printed on white paper and (**b**) spectral reflectance for colours measured on the colour monitoring ruler. Measurement of white was conducted on a white calibration plate. (source: authors).

Figure 2(a) is a colour monitoring ruler (printed on white paper) that was designed to serve as a reference while capturing the images of test samples. Colours on the monitoring ruler were measured using a handheld spectrophotometer. Their spectral reflectance are shown in Fig. 2(b). Spectral reflectance indicates the intensity of reflected light at a tested wavelength for the surface of an object. The white and black colours showed nearly consistent reflectance within the visible light spectrum. A white surface reflects most of the light, whereas a black surface has high absorbance. Blue and red colours have high reflectance at 400–500 and 600–700 nm, respectively. Yellow colour reflects light at a broad range (500–700 nm), whereas magenta colour absorbs light at 500–600 nm.

A handheld spectrophotometer normally has a small aperture of 8 mm unless specified otherwise^15^. Therefore, the colour of a surface is typically determined using the mean of a few measured spots. For example, on a 100 mm × 100 mm surface, 6 and 15 measurements were captured by Arruda Junior et al.^32^ and Jesus et al.^15^, respectively.

**Fig. 3:**
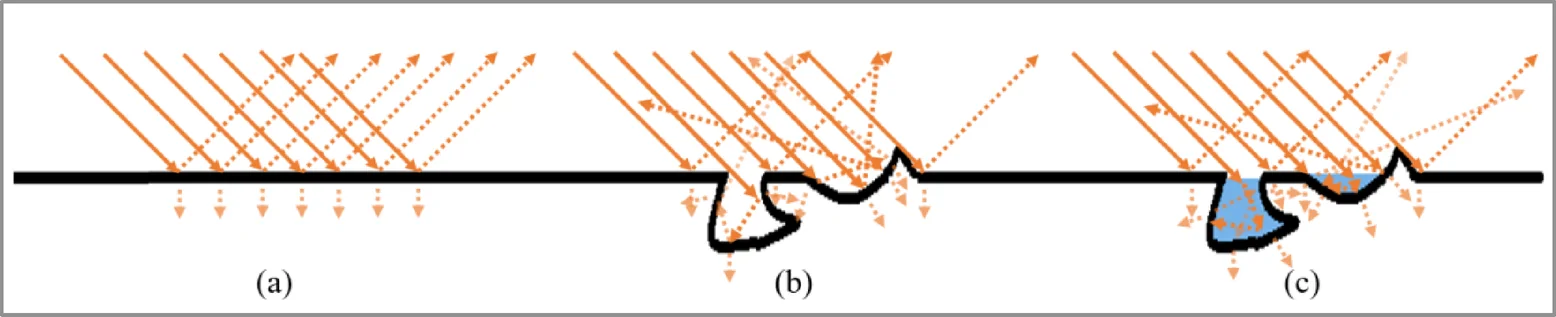
Absorption, reflection and refraction of light on (**a**) flat surface, (**b**) porous and uneven dry surface, and (**c**) porous uneven surface with moisture. (source: authors).

Notably, hardened CBMs’ surface conditions, such as pores of different sizes, uneven texture and moisture on the concrete surface, affect the results of colour measurements. In these conditions, the results of colour measurements vary because of differences in the levels of reflected, refracted and absorbed light^14,33^. Figure 3(a) illustrates light reflection on a flat surface, where part of light (colour) was absorbed by an object. Figure 3(b) illustrates how the presence of pores and protruded uneven surfaces scatter light towards different directions, facilitate the absorption of light by the object and consequently reduce the intensity of reflected light. When light passes through a solution, the solute and solvent in a solution may transmit, absorb, reflect, refract and scatter light that will degrade the quality of the spectrum^34^. The moisture (solution) in pores alters light scattering and yields a dark surface, as illustrated in Fig. 3(c). In addition, uneven surface can cause ambient light to leak into the test area when performing colour measurements using a spectrophotometer. Therefore, for a more accurate measurement of the surface colour, the test point shall be flat and dry and the proportion of pores within the millimetre-scale measurement area should be minimised.

A viscous cementitious mixture without the use of chemical admixtures is difficult to properly fill into the formwork. Chuta et al.^7^ cast an OPC paste mixture with w/c ratio of 0.30 in a 40 mm × 40 mm × 160 mm metal mould; they observed many large pores (4 mm) on the formed surface of the hardened paste, but the number and size of the pores decreased with increasing w/c ratio. Czarnecki and Sadowski^35^ observed that the total volume of voids decreased in hardened OPC paste with w/c ratio of 0.50 relative to the total volume obtained at w/c ratio of 0.35 that cast with a 150 mm cubical mould. Void volume on an OPC paste surface is affected by the type of mould. A mould made from an oriented strand board has the highest void volume, followed moulds made from wood, steel and plastic^35^. These studies indicate that w/c ratio and mould type affect the appearance of the formed surface for cement paste and should thus be considered in the colour measurements of CBM surfaces. Furthermore, different combinations of different mould types and release agents also affect the surface void (including size and distribution), roughness and colour variation of the concrete surface^36–37^. Other factors, such as compaction methods and addition of superplasticizer that influence the workability of concrete and/or mortar, may affect their surface appearance. However, relevant literature is limited.

For air-cured samples, whether in an ambient environment or in a chamber with controlled temperature and humidity, colour measurements can be conducted directly without an extra drying step^3,38^. Chuta et al.^7^ found that curing in air with temperature of 20 °C and relative humidity of 45%, the surface colour stabilised after 4 days for paste and 2 days for mortar. However, for water-cured samples, colour measurement cannot be conducted until the influence of moisture is minimised. To prevent the influence of moisture during colour measurement, Assaad et al.^39^ oven-dried specimens at 50 ± 5 °C for 1 day, and Yamaguchi et al.^40^ air- dried samples before testing.

The instantaneous complete image of a flat surface can be recorded using either a camera or a flatbed scanner, but both tools are limited. A camera can capture a high-precision image, but the angle must be carefully adjusted to reduce the effect of ambient lighting, which can cause shadows or overexposed area^14^. A flatbed scanner is useful for capturing pore distribution on a concrete surface for analysis^41^, but uneven areas that are not in close contact with the scanner will be out of focus, resulting in unclear images.

### Research materials and methods

The sub-surface of a hardened concrete can be divided into cement skin (0.1 mm), mortar skin (5 mm) and concrete skin (30 mm); cement skin is cement paste that contacts the formwork and demoulding agent^35^. Chuta et al.^7^ reported that the colours of the grey paste and mortar’s surfaces lighten as the w/c ratio increased. In addition, the surface reflectance of a concrete is predominantly influenced by the reflectance of the cement, followed by sand^8^. Therefore, the focus of this research was on the cement paste. OPC and WPC were replaced with moderate (30%) and high-volume (50% and 70%) GGBS, and the effects of the replacement on the surface colour changes of pastes in the presence and absence of red iron oxide (IO) pigment was examined. The GGBS with off-white colour was used because it is a common SCM with a light colour^42^ and can be used in high-volume replacement with good engineering properties of hardened CBMs^43–44^.

The influences of surface texture conditions and chemicals on the evaluation of chromatic properties were minimised by casting the samples into plates with smooth surfaces without release agent and superplasticizer. The same water content was used for all mixtures.

### Materials and mix proportions

The study was conducted in four series of cement pastes: (1) OPC-based mixture, (2) WPC-based mixture, (3) red IO OPC-based mixture and (4) red IO WPC-based mixture. Red IO pigment is commonly used as an integral pigment for CBMs because of its good resistance to light (not easily faded), high opacity, inertness to cement, non-toxicity, low cost and abundance^45–46^.

**Fig. 4:**
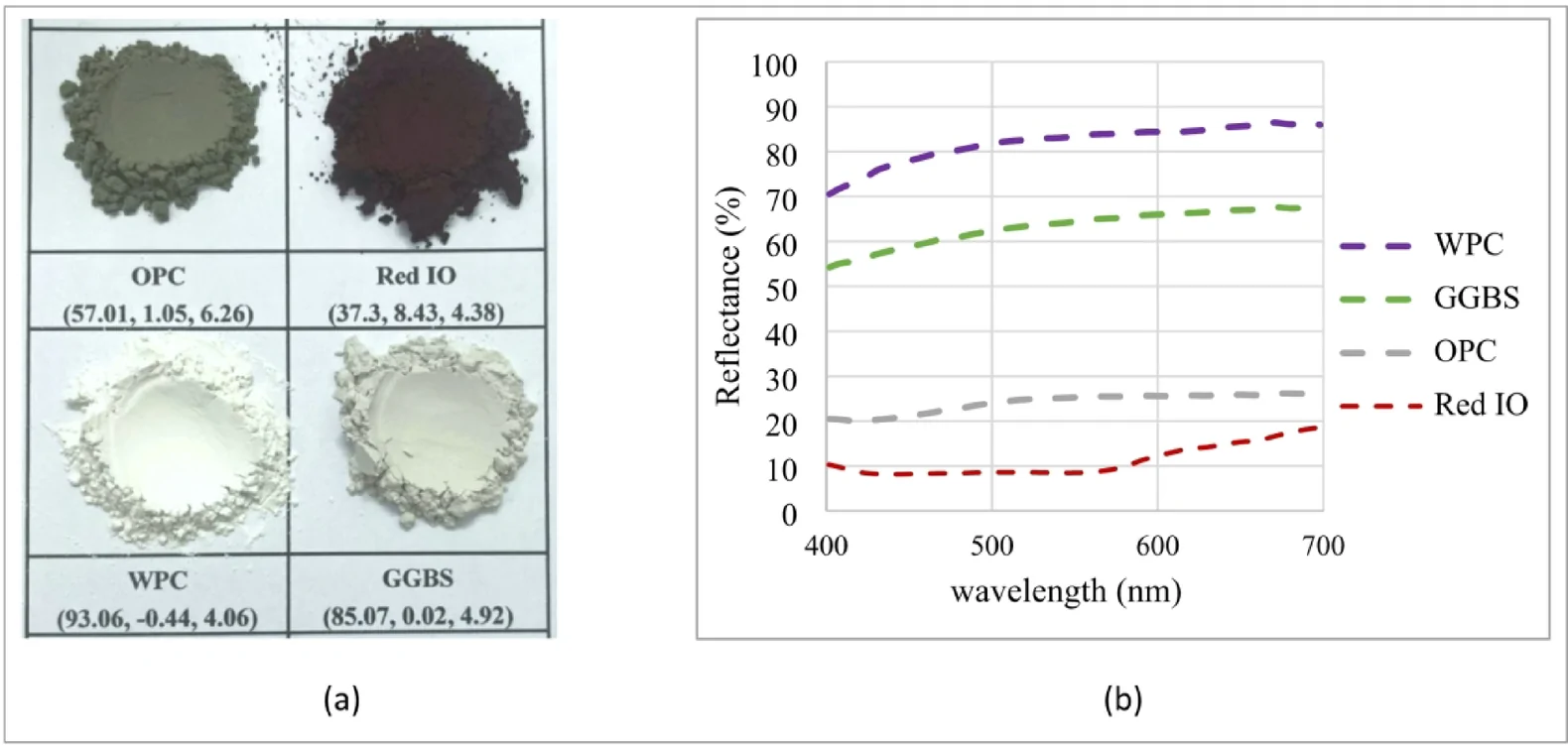
(**a**) Physical appearance and (**b**) spectral reflectance of OPC, WPC, GGBS and red iron oxide (IO) powder. The number in the brackets denotes L*, a* and b* of the CIELAB colour for each material.

The binder used in this study were OPC (CEM I 42.5 N), WPC (CEM I 52.5 N) and GGBS, with densities (tested according to ASTM C188^47^ of 3.12, 3.02 and 2.86 g/cm^3^, respectively. The chemical composition of materials used are listed in Table 1. The negative value in loss on ignition for GGBS is attributed to the oxidation of sulphides^48–49^. Figure 4(a) shows the physical appearance of the materials. The spectral reflectance of the powders was tested using a spectrophotometer and the result is shown in Fig. 4(b). As shown in Fig. 4(a), the red IO is darker than the OPC. Figure 4(b) shows that the spectral reflectance of red IO is lower (7.2%–16.8%) than OPC across the visible wavelength.

**Table 1:** Chemical compositions and loss on ignition (LOI) of raw materials (% by mass).

Compositions	CaO	SiO_2_	Al_2_O_3_	MgO	Fe_2_O_3_	SO_3_	MnO	LOI
OPC	63.82	19.27	5.76	2.20	4.54	3.00	0.16	1.2
WPC	71.18	17.95	3.65	0.95	0.32	4.70	-	3.4
GGBS	41.65	30.71	14.33	7.91	0.66	1.90	1.02	-0.76
Red IO	2.75	3.42	2.58	-	83.69	0.09	0.21	9.14

Sixteen cement paste mixtures were prepared (Table 2). The cement paste was prepared using a constant water content. At this water content, all mixtures could be thoroughly mixed without producing excessive bleeding in the most fluidic paste (i.e. O-Ctrl) or becoming too stiff in the paste with highest viscosity (i.e. R-W-G70). The GGBS replacement for cements was based on volumetric ratio, and the pigment was added at 5% by weight of cement.

**Table 2:** Mix proportions of the cement paste mixtures in one batch.

Series	Mixture	Binder (g)			Red IO (g)	Water (g)
		OPC	WPC	GGBS		
OPC-based mixture	O-Ctrl	2000	-	-	-	560
	O-G30	1400	-	550	-	560
	O-G50	1000	-	917	-	560
	O-G70	600	-	1283	-	560
WPC-based mixture	W-Ctrl	-	2000	-	-	560
	W-G30	-	1400	568	-	560
	W-G50	-	1000	947	-	560
	W-G70	-	600	1326	-	560
Red IO OPC-based mixture	R-O-Ctrl	2000	-	-	100	560
	R-O-G30	1400	-	550	100	560
	R-O-G50	1000	-	917	100	560
	R-O-G70	600	-	1283	100	560
Red IO WPC-based mixture	R-W-Ctrl	-	2000	-	100	560
	R-W-G30	-	1400	568	100	560
	R-W-G50	-	1000	947	100	560
	R-W-G70	-	600	1326	100	560

### Casting and curing of specimens

Cement paste was prepared according to MS EN196-3^50^. Dry powders were added to the 5 L mixer bowl with water gradually. This method allowed cement particles to come in contact with water. At a low speed, the mixture was mixed for 1.5 min, mixing was then stopped for approximately 45 s and paste that adhered to areas outside the mixing zone was scraped back into the mixing section with a silicone spatula. The mixing continued for another 1.5 min at a low speed.

The mixtures were cast into 100 mm × 100 mm × 1.5 mm plate specimens using polypropylene moulds. To remove the air bubbles on the mixture, the freshly moulded pastes were compacted by tapping the tabletop with the mould manually for approximately 100 times within 20 s. No release agent was applied on the mould during casting. The fresh pastes were covered with plastic sheets to prevent the evaporation of moisture and were demoulded after 24 h. Four specimens from each mixture were used. For each mix, two specimens were air-cured (A) in the laboratory environment with a temperature of 31 °C ± 2 °C and relative humidity of 70% ±5%. Another two specimens were water-cured (W) for seven days in saturated lime water at a water temperature of approximately 29 °C. During water curing, the specimens were laid flat without stacking to prevent scratching by another specimen and to ensure uniform salt deposition on the surface. On day 7, water-cured samples were washed with pipe water and lightly wiped, and loose white powder (calcium carbonate forms on the surface due to saturated lime water) on the surface of the specimens was removed. The specimens were then air-cured until the testing day.

**Fig. 5:**
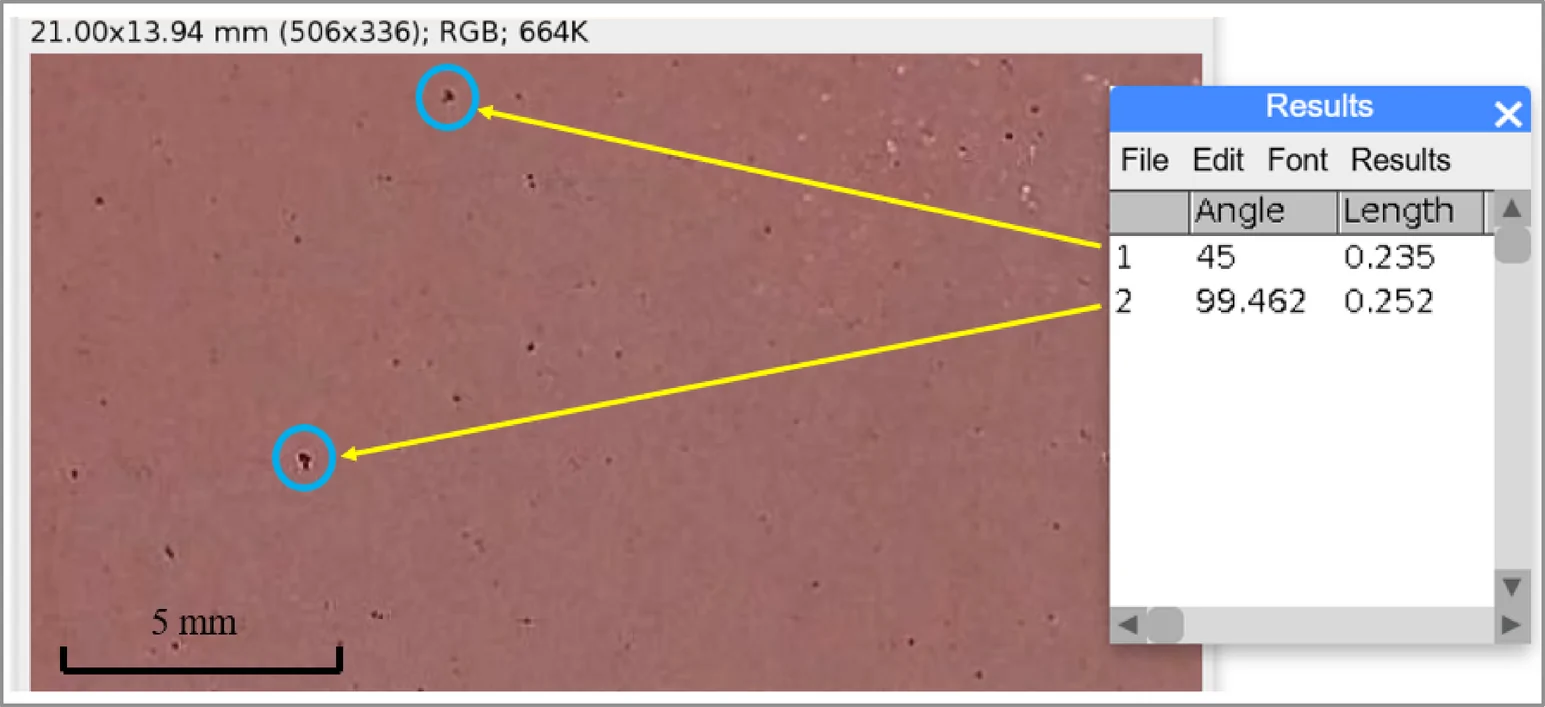
Pore size measurement (in mm) using ImageJ software.

A visual inspection on the formed surface of all specimens in this study showed no evident voids and colour inconsistency on the surface. Such appearance was better than that observed in samples cast by Czarnecki and Sadowski^35^ and Chuta et al.^7^, although the selected w/b ratio was less in current study. The reasons may be the used mould was made from polypropylene with a smooth surface and the thickness of samples was only 1.5 mm, which allowed for the escape of air trapped in the mixture through manual compaction. Randomly distributed pores with a diameter less than 0.5 mm were observed on the surfaces of the specimens through ImageJ software (Fig. 5). Therefore, the prepared specimens met the basic requirements for visual observation and colour assessment, analysis of surface colour in scanned images and colour measurement with a spectrophotometer.

### Testing method

**Fig. 6:**
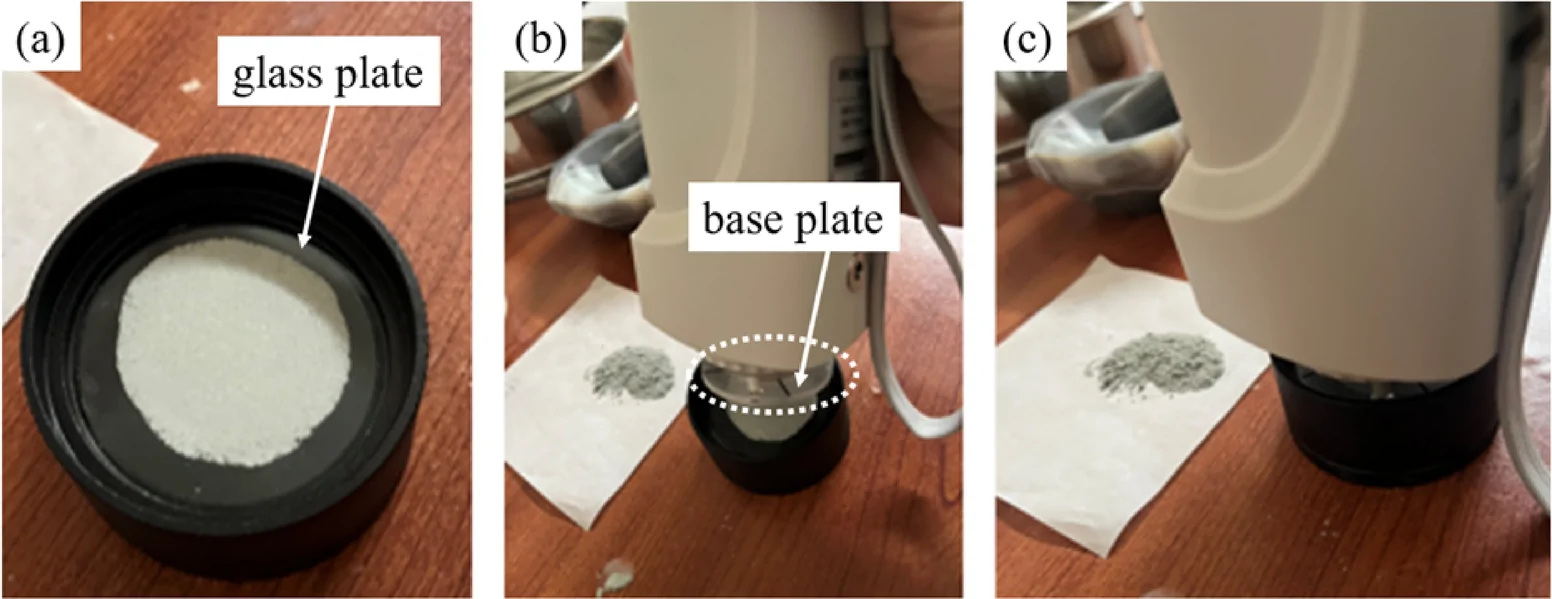
(**a**) Powder holder with a sample powder spread evenly below the glass plate for colour measurement with a handheld spectrophotometer, (**b** & **c**) the aperture at the base of the spectrophotometer was in full contact with the glass plate. This setup prevents light leakage during measurements.

The chromatic properties of the powder (before hydration) and hardened cement pastes were measured using a handheld spectrophotometer. This device is with a 40 mm integrating sphere, 8° viewing angle, covering 400–700 nm range at 10 nm wavelength pitch. The specular component included (SCI) mode and a D65 standard illuminant was set at a standard observer angle of 10° and measurement was performed through an 8 mm aperture. In the SCI mode, spectral reflectance from specular and diffused light are measured. To prevent light leakage during measurement, the spectrophotometer aperture should be in close contact with and perpendicular to the specimen surface. A wide base plate installed near the aperture (Fig. 6(b)) can facilitate this. For powder measurement, a powder holder with a thin glass is required to prevent contamination of fine powder through the aperture into the integrating sphere. For an accurate measurement, the powder shall be filled until an even colour flat layer formed under the thin glass, with a diameter not less than 8 mm from the centre of the holder (Fig. 6).

**Fig. 7:**
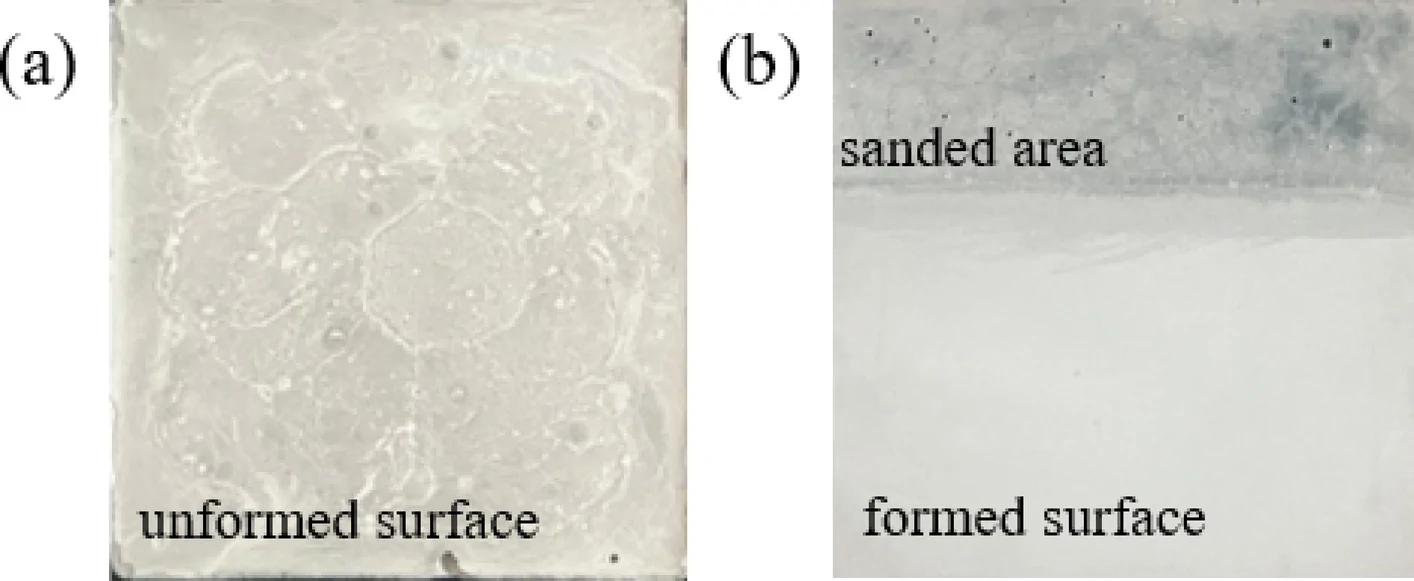
(**a**) Uneven texture and colour of the unformed surface of cement paste samples. (**b**) formed surface with and without slight-polishing.

The chromatic properties on 100 mm × 100 mm surface of specimens were measured at the ages of 1, and subsequent 7 days gap at 8, 15, 22 and 29 days. For a thin specimen (with a thickness of 15 mm in this study) there are two available surfaces for the colour measurements, the unformed surface (the exposed top face) and formed surface (the exposed bottom face to the mould). The formed surface was selected for testing because of three reasons: (1) the exposed concrete appeared on the formed surface (except in flooring application), (2) the surface texture of an unformed surface was not flat or smooth unlike the formed surface and (3) colour uniformity on the top surface was lower because of bleed water (Fig. 7(a)). Nevertheless, some studies conducted measurement on the unformed^51^, side^38^, or cut surfaces^39^. Trial study on slightly-polished hardened paste surfaces using sand paper showed increased porosity and uneven colour (Fig. 7(b)). The occasional blue hue (top right corner of Fig. 7(b)) was attributed to the GGBS content in the paste mixture, and this hue may fade over time.

Before the colour measurement with a spectrophotometer, the surface of the specimen was lightly wiped to remove loose fine particles that may damage the instrument^14^. Four measurements were obtained from the surface of each specimen from each quadrant. Two specimens were measured from each mixture. The used colour space was CIELAB, and the calculation for ∆*E*_00_ was performed using a spreadsheet provided by^28^. In addition, a flat-bed scanner was used to record the overall appearance of the specimen at 600 dpi.

## Results and discussion

### Visual colour assessment of binder-powder blends

**Fig. 8:**
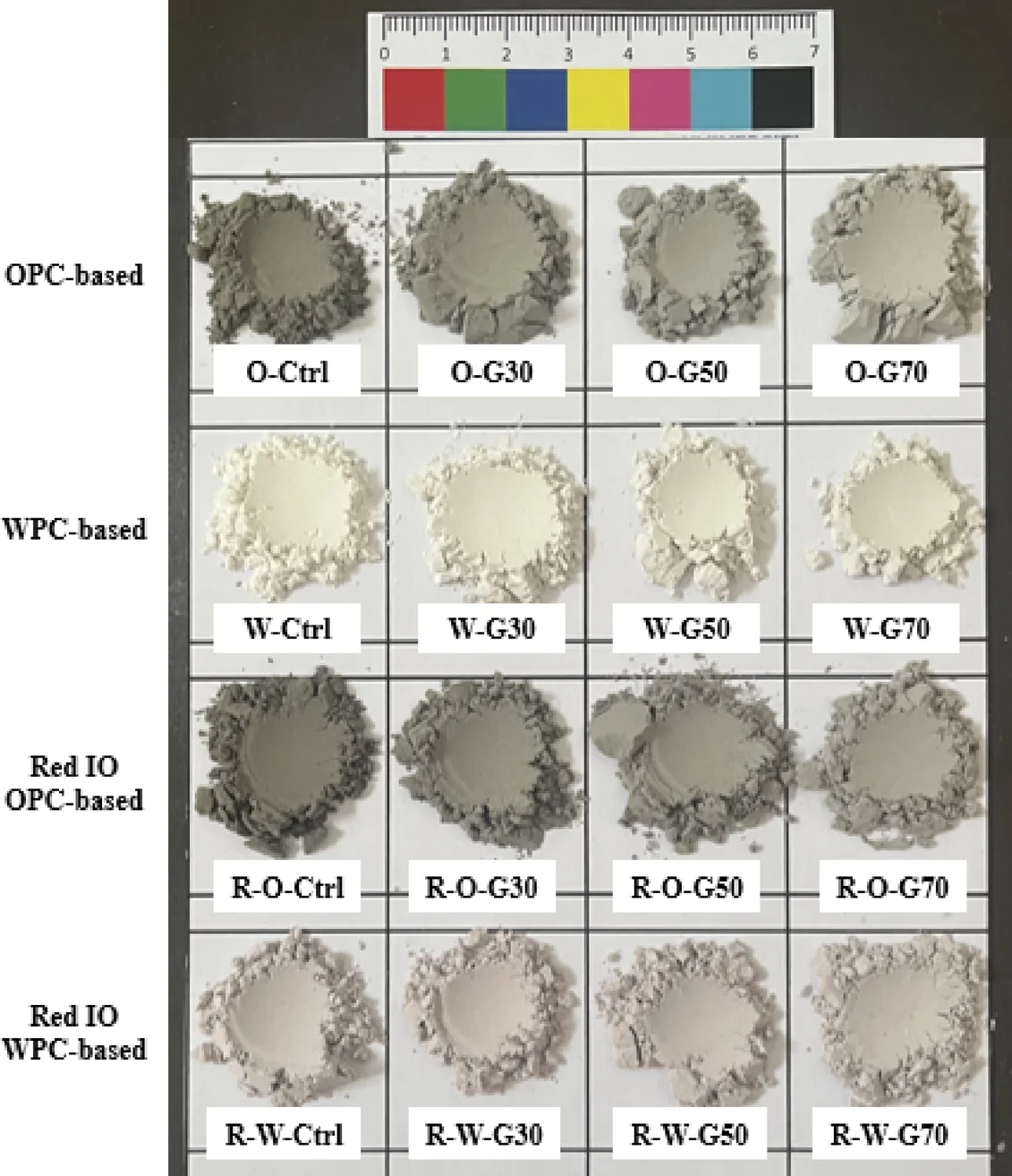
Colour of all mixtures before the presence of water.

Eight unpigmented and eight pigmented paste mixtures were prepared in this study. Figure 8 shows the colour of all these mixtures before water was added. The first (leftmost) columns are control mixtures (without GGBS), and the second, third and fourth columns are the blended powders containing 30%, 50% and 70% GGBS, respectively. After the addition of 30%–70% GGBS, the OPC-based mixture showed a lighter colour, whereas the WPC-based mixture showed a duller appearance (Fig. 8). The addition of red pigment powder was not evident in the red IO OPC-based mixture, whereas the red IO WPC-based mixture showed a faint pink colour. Although the presence of pigment was visually less prominent in dry mixes than in wet mixes, its colouring effect becomes evident in the presence of water. This observation is consistent with that of Jinnai^52^ when yellow, blue, and green pigments were mixed with WPC at replacement ratios of less than 10%. The pigments produced light-yellow, light-blue and light-green colours dry mixtures.

### Visual colour assessment of hardened pastes

**Fig. 9:**
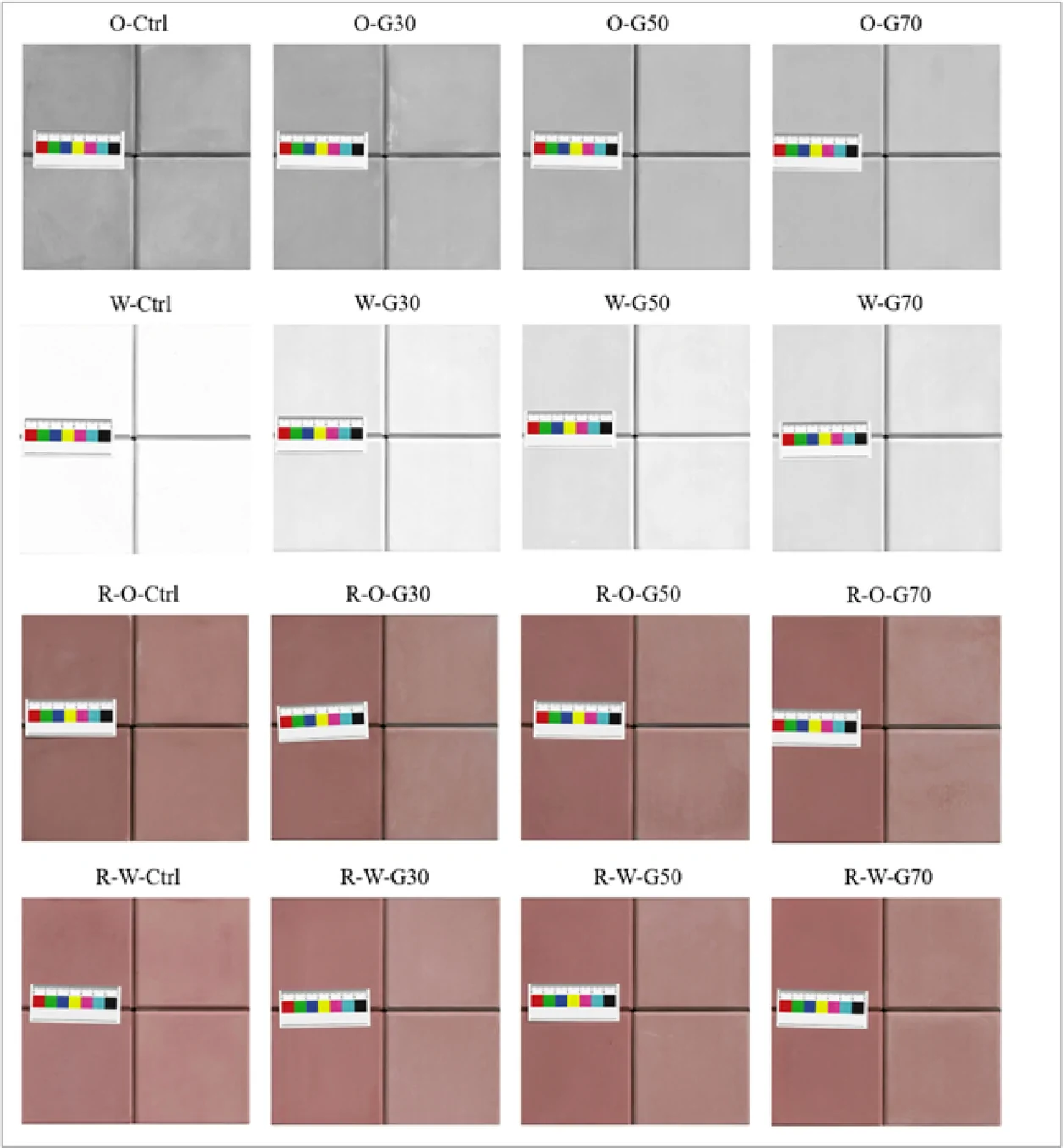
Scanned images for different mixtures subjected to air-curing (left column) and water-curing (right column) on day 29.

Figure 9 shows the images of all mixes at the age of 29 days. The images are the result of scanning four specimens (plates) for each mix, during which two plates were air-cured (arranged on the left column) and the other two underwent water-cured (arranged on the right column). The water-cured samples were lighter in colour than the air-cured samples. For the OPC-based mixes, the grey colour becomes lighter and more homogeneous as the GGBS replacement ratio increases. As for the WPC-based mixes, the white surface became increasingly greyish. A comparison between the colour of OPC- and WPC-based mixes showed that irrespective of curing type, the difference between the colours of the two series became less evident as GGBS content increased, although difference between O-G70 and W-G70 was still observed. However, after the addition of red IO pigment to the OPC- and WPC-based series, the colour difference between the two groups became negligible.

The scanned images of pigmented specimens shows that the pigment was well distributed in the mixtures, and the colour difference became less visible regardless of GGBS ratio in each series. Moreover, the samples of the WPC-based red mixture were brighter than the OPC-based red mixture. However, this brightness decreased with increasing GGBS content. This observation is consistent with the observation of Jang et al.^53^, who studied on red pigmented WPC mortars with different GGBS replacement ratios (0%, 20%, 40% and 60%). From the photos of the specimens in that study, it could be observed that there are random patches of white efflorescence on the mortar surfaces which may affect the results of colour measurements. However, they did not mention whether an area was avoided during the sampling for measurement spots.

**Fig. 10:**
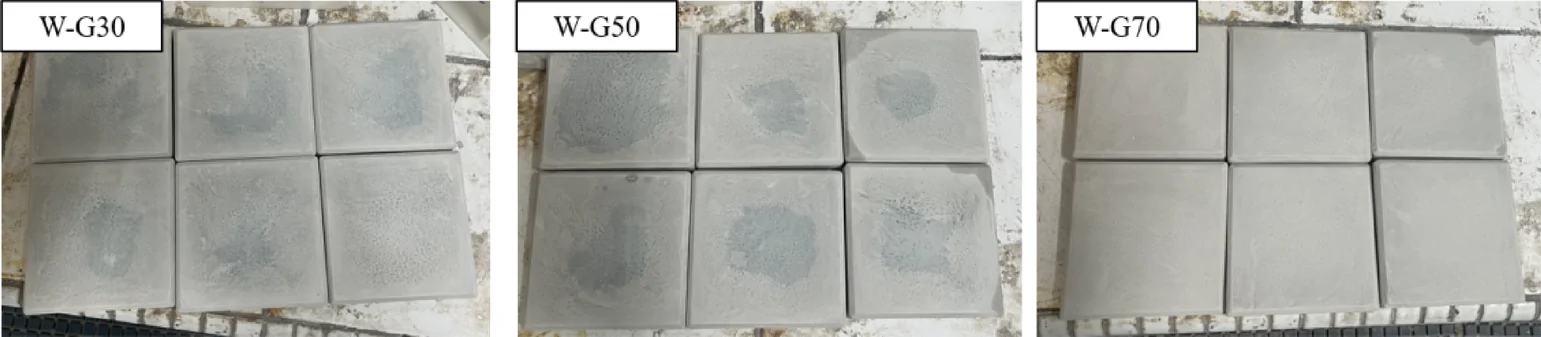
W-G30, W-G50 and W-G70 specimens immediately after demoulding.

Inconsistent green-blue discoloration might appear in hardened cement mixtures containing GGBS right after demoulding^54^. It usually occurs when sulphides in GGBS react chemically with compounds in cement with the absence of air between the hardened surface and mould^55^. In this study, the green-blue discoloration shown in Fig. 10 was clearly visible in the W-G30 and W-G50 cement paste immediately after demoulding. This discoloration gradually subsided within the first 7 days after demoulding because the green-blue sulphides on the surface were oxidised to form colourless compounds upon exposure to air^54–55^.

### Colour uniformity study using CIELAB colour difference

**Fig. 11:**
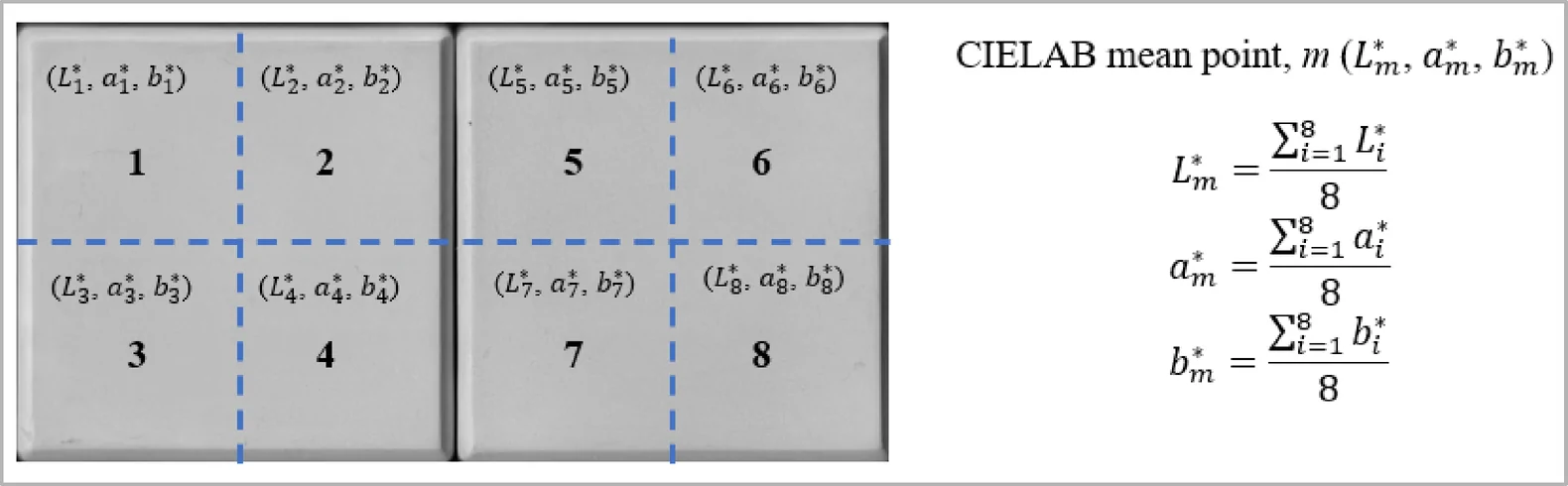
Measured and mean points (calculated using the formulas provided) for CIELAB colour measurement using a handheld spectrophotometer.

Apart from visual inspection, colour uniformity can be determined using the colour difference ($\:{\varDelta\:E}_{00}^{im}$) between the measured point (*i*) and mean point (*m*)^25^. Figure 11 displays the sampling of colour measurement for two specimens from each mixture, where one measurement for each quadrant was conducted for each sample. The mean point was calculated from the mean of each component in the CIELAB space, as demonstrated in Fig. 11. In the ideal case, the ∆*E* should be zero where the surface has a uniform colour^29^.

**Fig. 12:**
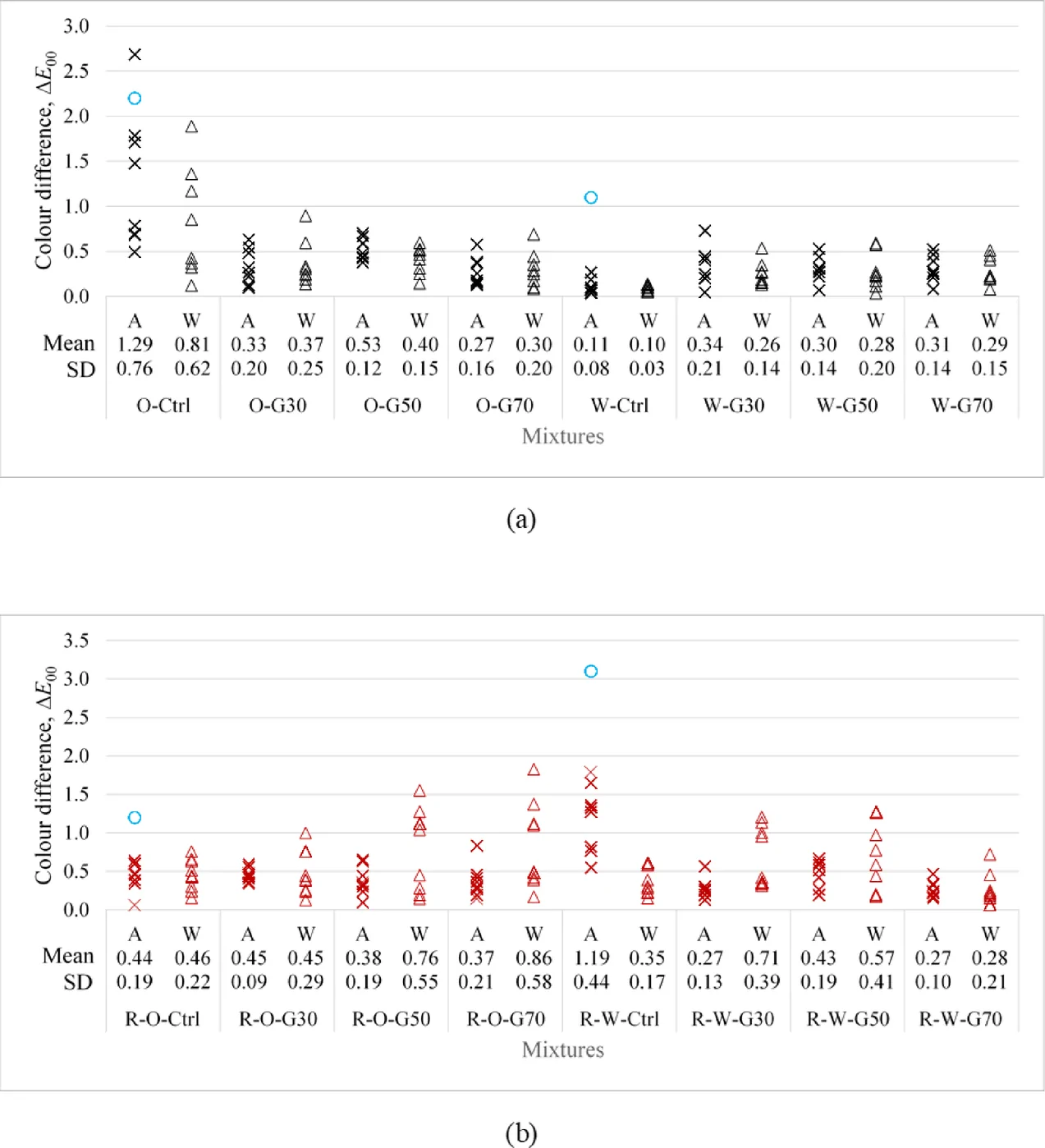
Distribution of colour difference (∆*E*_00_) between the measured points and mean point of air-cured (A) or water-cured (W) specimens for (**a**) unpigmented and (**b**) pigmented 29 days pastes. The mean and standard deviation (SD) for each mixture is presented at the x-axis. × for air-cured specimen, ∆ for water-cured specimen and ○ is the mean value for similar mixtures from^51^.

Figure 12 shows the $\:{\varDelta\:E}_{00}^{im}$ value for air-cured (A) and water-cured (W) for mixtures. Eight spots for each mix was measured. The mean and standard deviation (SD) of ∆*E*_00_ for each mixture is presented at the x-axis. This figure was provided to present the distribution of the ∆*E*_00_ for each mix that reflects the degree of colour uniformity. Small mean and SD values indicate a uniform colour on a sample surface. Given that the influence of surface porosity and texture is minimised for this batch of specimen, the uneven colour may be due to the intrinsic chemical properties of the mixture. The main colouring component in O-Ctrl was Fe_2_O_3_, and only about 5% of Fe_2_O_3_ in the OPC content. Such a low level of colouring component fails to distribute uniformly in the cement paste.

Figure 12 shows that among the air-cured mixes, W-Ctrl had the highest surface uniformity (mean ∆*E*_00_ = 0.11 ± 0.08), and the O-Ctrl had the lowest (mean ∆*E*_00_ = 1.29 ± 0.76). However, when red pigment was added, the opposite was observed. The mean ∆*E*_00_ values for R-W-Ctrl and R-O-Ctrl were 1.19 ± 0.44 and 0.44 ± 0.19, respectively. The colour uniformity of the unpigmented paste was generally higher than that of the pigmented paste, except O-Ctrl. The air-cured samples showed better colour uniformity than water-cured samples, with an average ∆*E*_00_ values of 0.39 and 0.52, respectively. The colour uniformity for all mixes was observed visually (Fig. 9). Figure 12 correlates to the findings reported by López and Sarli^51^, where (1) ∆*E*_00_ for WPC mortar is smaller than OPC mortar at 1.1 versus 2.2 and (2) ∆*E*_00_ of WPC-based red mortar is larger than OPC-based red mortar at 3.1 versus 1.2.

### Colour monitoring during curing period

When the specimens were demoulded 24 h after casting, the surface colour of the formed surface may not be uniform (Fig. 10) compared with the appearances occurring on day 8 and onwards. The water-cured samples were removed from the water on day 7, and colour measurement was conducted 1 day after drying. This step was performed to minimise possible errors due to the influence of dampness near the surface. Compared with a dry surface, a moist surface has more and larger dark spots because of the influence of moisture inside pores and cracks. Therefore, there should be no visible surface moisture during colour measurements^56^.

**Fig. 13:**
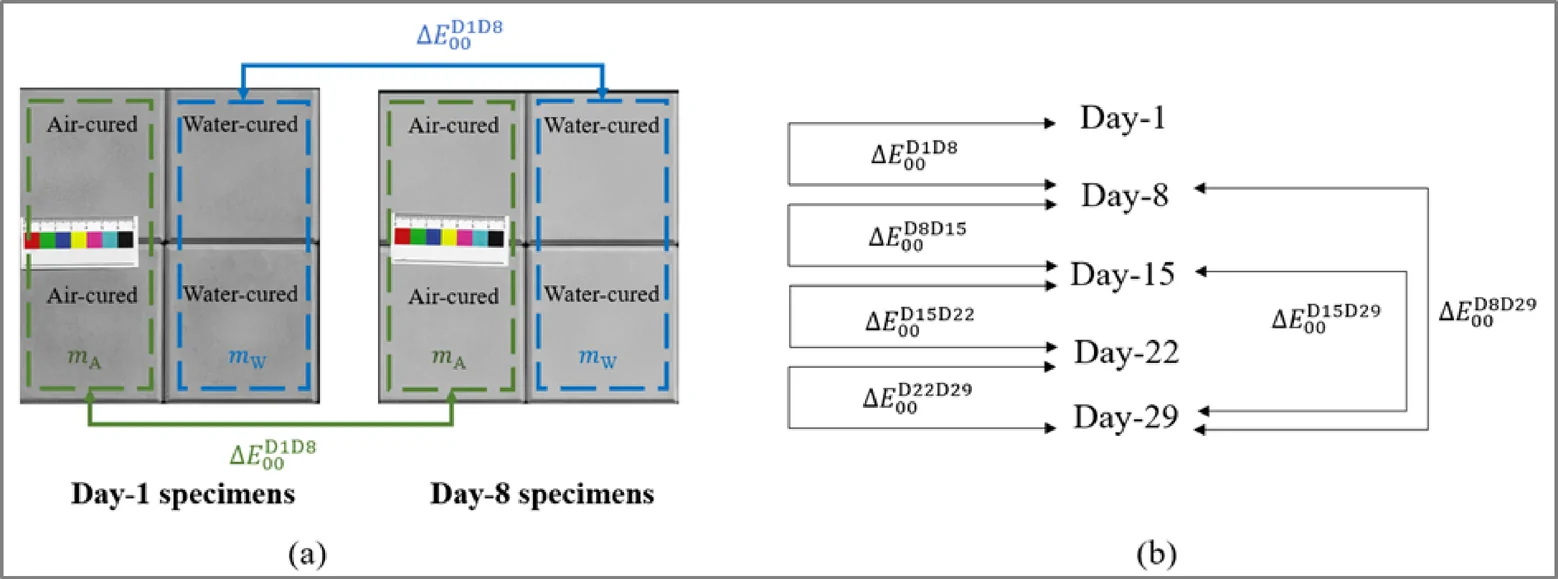
Colour difference (∆*E*_00_) monitoring for (**a**) air-cured (A) and water-cured (W) samples. (**b**) The comparison between days 1 and 8 is denoted as D1D8. Other comparisons were designated in the same manner.

**Fig. 14:**
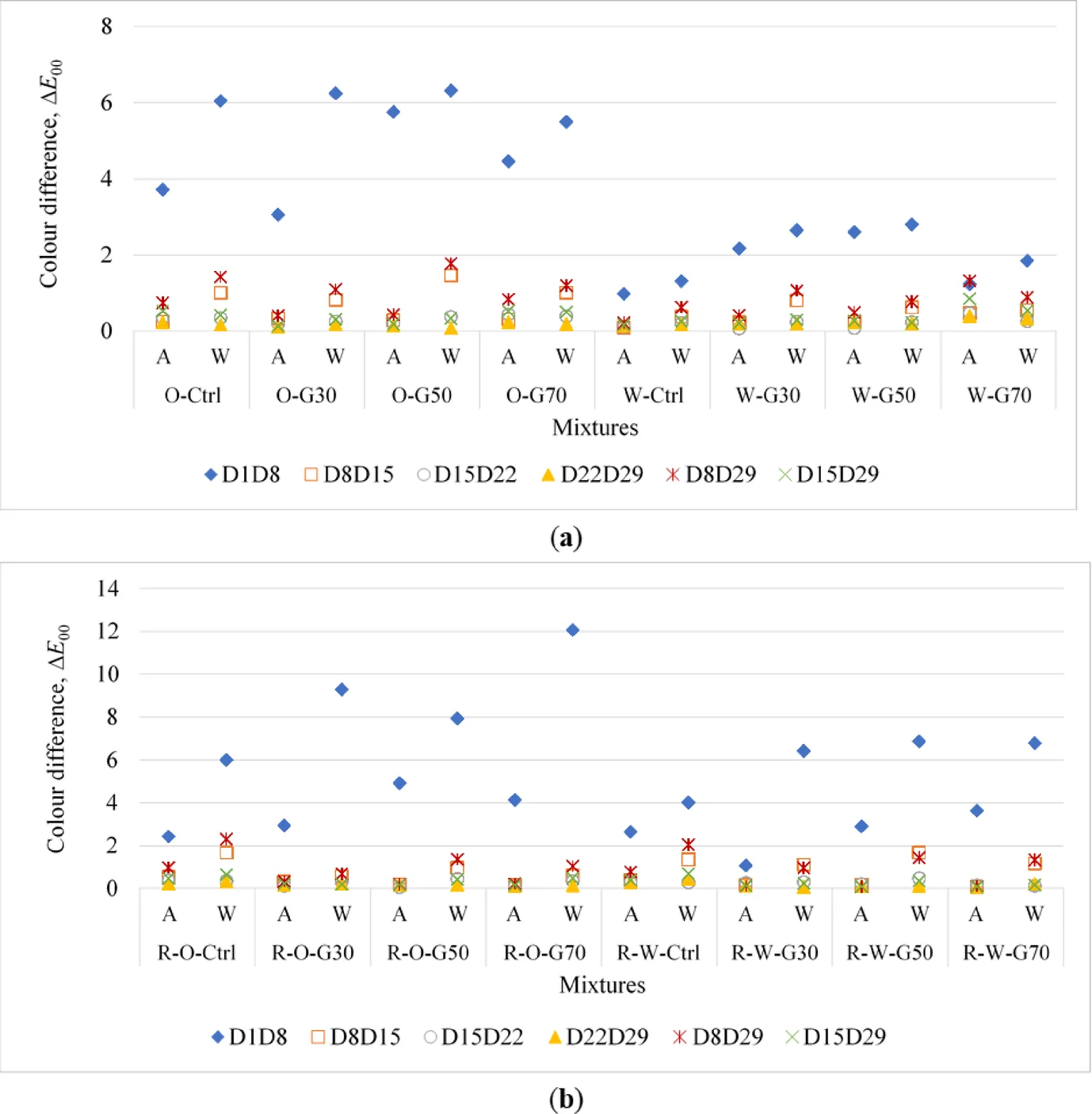
Colour difference (∆*E*_00_) values for air-cured (A) or water-cured (W) (a) unpigmented and (b) pigmented specimens (D1D8 is the colour difference between days 1 and 8 samples).

Figure 13 illustrates the method for determining the colour difference between specimens in different ages under air-cured (A) and water-cured (W) conditions. The degree of colour difference was examined using different reference periods. Each mixture is presented by a CIELAB mean point (Fig. 11). Figure 14 reveals that the greatest colour change occurred between days 1 and 8 $\:(\varDelta\:{E}_{00}^{D1D8})$ irrespective of curing condition. However, the value for a WPC specimen was considerably lower than that of an OPC specimen at the same GGBS replacement level. For the water-cured samples, the $\:\varDelta\:{E}_{00}^{D8D15}$ and $\:\varDelta\:{E}_{00}^{D8D29}$ values were about 2, remaining high and indicating that 1 day drying for the water-cured specimens (samples were water-cured until the age of 7 days and then followed by air-cured condition) may not be sufficient for colour measurement given that the surfaces of samples contain moisture. The ∆*E*_00_ values after 15 days (D15D22, D15D29 and D22D29) were small (less than one), showing that colour to stabilises at least 7 days after demoulding or removal from water. Therefore, colour assessment can be conducted any time after 7 days of exposing the specimens into air (under ambient condition). It was also observed that there is no obvious difference in the drying rate between cement paste with and without pigment.

**Fig. 15:**
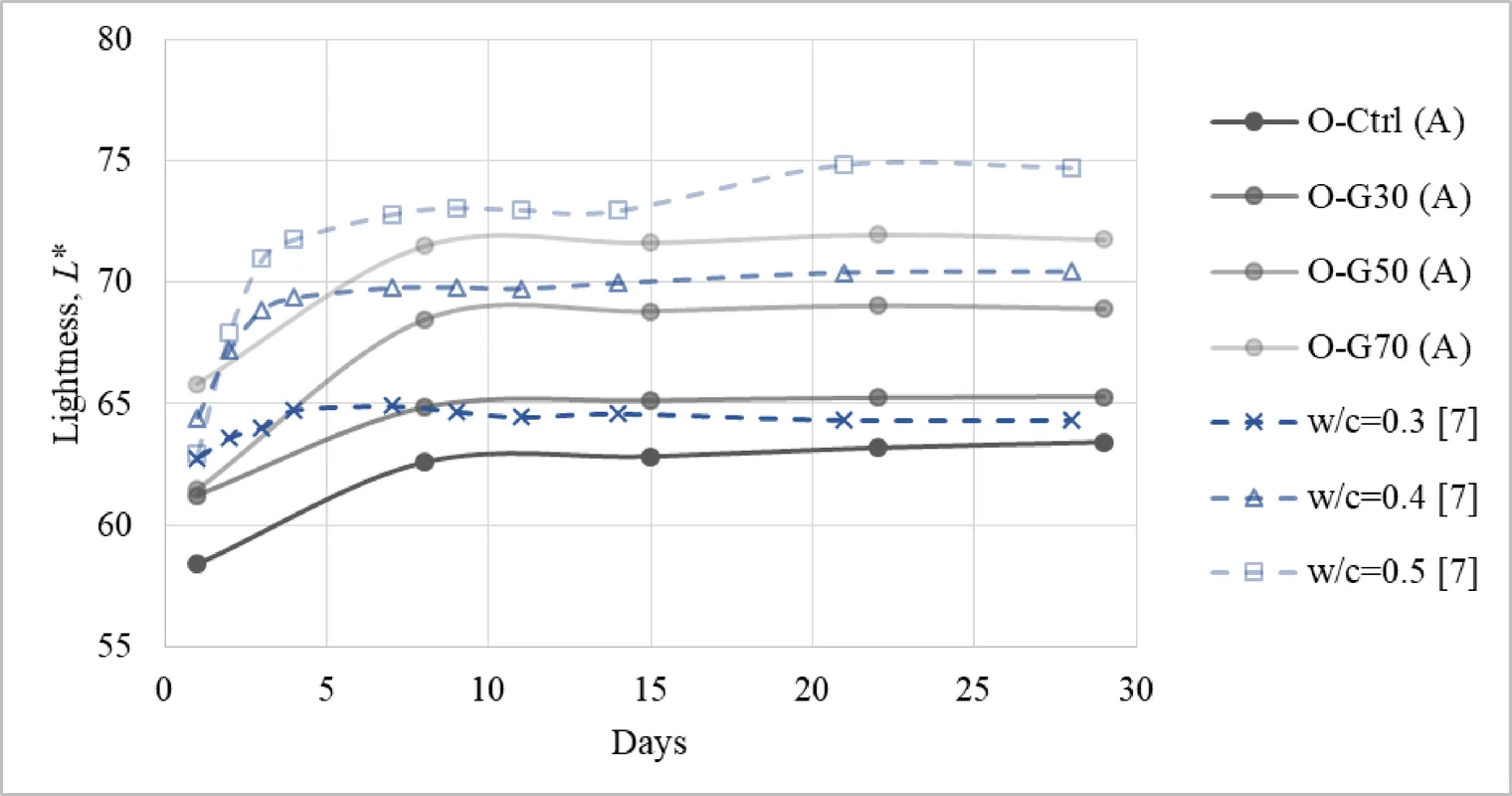
Changes in the surface lightness (L*) value of OPC paste cured in air in the current study and^7^.

Chuta et al.^7^ monitored the surface lightness (L*) of OPC pastes at different ages. The paste mixes had different w/c ratios (0.3, 0.4 and 0.5) and cured in the air after demoulding. Figure 15 shows a comparison between the results of the two studies. The results revealed that the lightness (L*) value increased by time. The increment was considerable on the first 3–7 days and then stabilised at the 20th day. Determining the age of the stabilised L* is important if the specimen will be used as a control sample for exposure tests. Figure 15 shows that a light surface for an OPC CBM can be achieved through two methods: by increasing the w/c ratio or by partially replacing OPC with a lighter colour SCM, such as GGBS. However, increasing the w/c ratio may not be a suitable solution because it negatively affects the mechanical and durability properties of CBMs. Many studies have demonstrated that incorporating of GGBS into cement mixtures improved these properties^57–58^.

### Effect of GGBS on colour difference

**Fig. 16:**
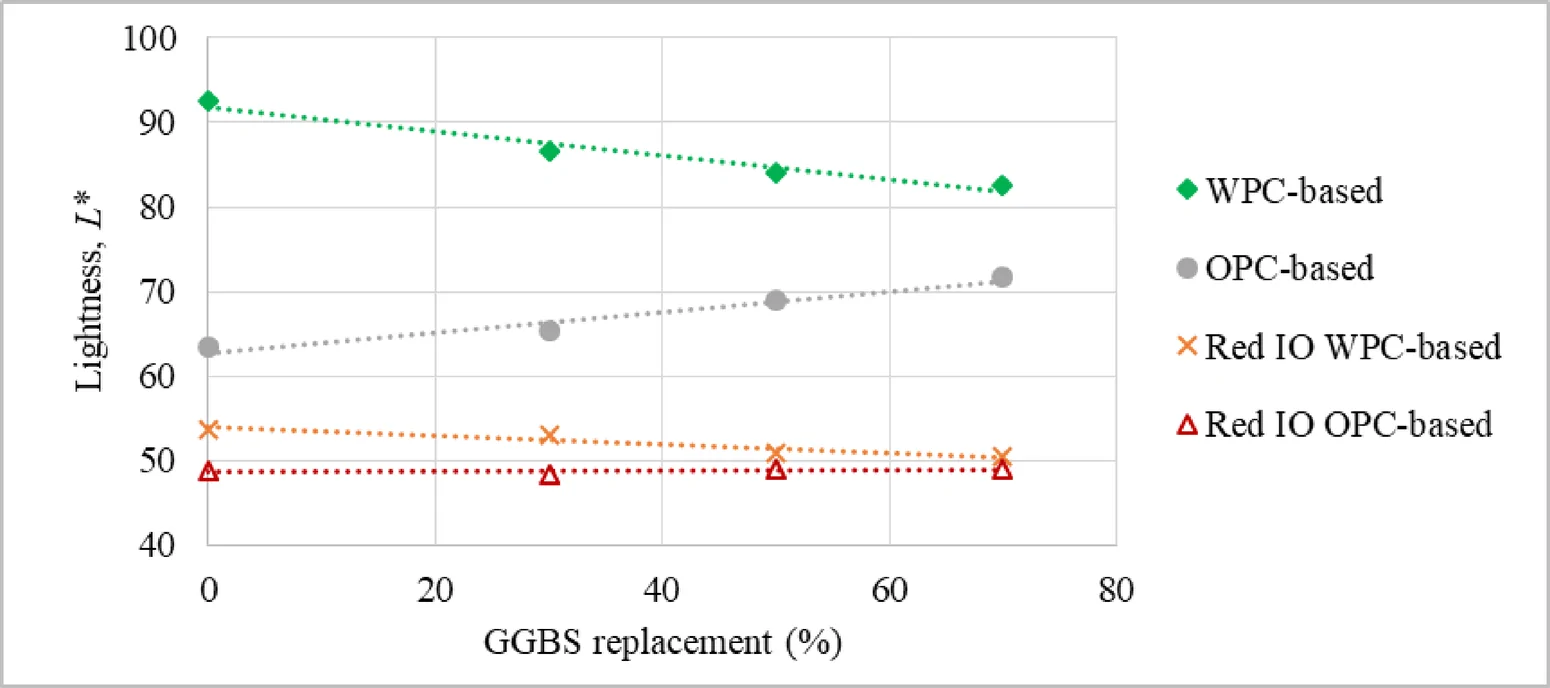
Relationship between the lightness (L*) of the surface of cement paste and GGBS replacement levels for air-cured specimens.

Figure 16 shows the surface lightness (L*) of cement paste (air-cured) with different GGBS content. As the GGBS replacement rate increased, the OPC-based mixtures became lighter in colour, whereas the WPC-based mixtures became darker in colour. The result is consistent with the findings of Schack et al.^59^, which showed a considerable increase in lightness when more GGBS was used. A highly correlated relationship was established on both series of unpigmented cement mixtures. The L* value of the cement paste changed at an average rate of about 0.13 per GGBS replacement ratio. However, in the red pigmented cement paste specimens, the L* value did not change with varying GGBS replacement ratio because of the influence of the red pigment, which had the lowest lightness (refer to Fig. 3(a)) among the raw materials exerting considerable effects on the final colour of the mixture.

**Fig. 17:**
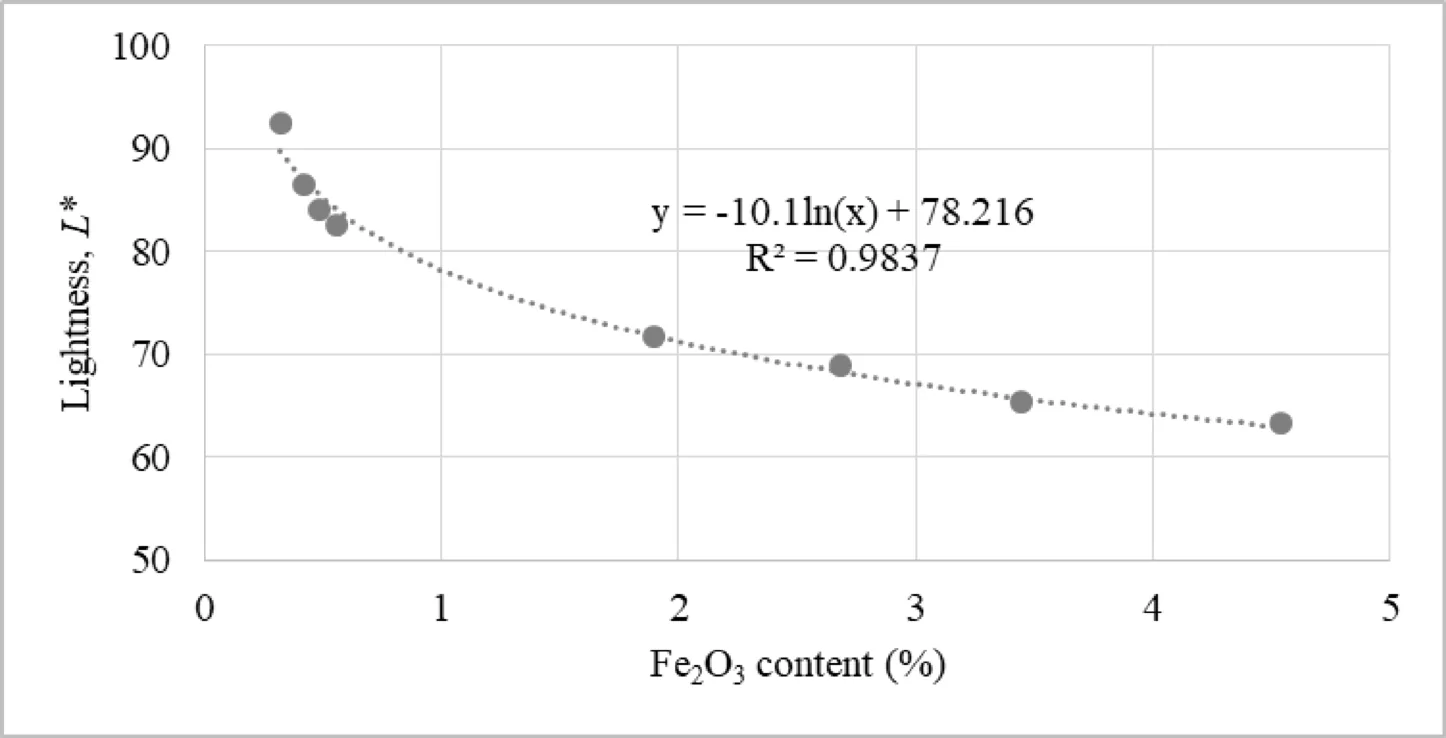
Correlation between surface lightness and total Fe_2_O_3_ content (%) for OPC- and WPC-based dry mixtures.

WPC is produced from raw materials with extremely low Fe_2_O_3_ and MnO contents, minimising the discolouration caused by these oxides while preserving the whiteness of WPC^60^. Table 1 and Fig. 4 exhibit the properties of the raw materials used in this study. The Fe_2_O_3_ content of the powder increased with the amount of absorbed light. Figure 17 shows the relationship between surface lightness and the total Fe_2_O_3_ content (%) of OPC- and WPC-based dry mixtures. A logarithmic relationship was observed for these unpigmented pastes with high correlation. This relationship may be useful in predicting the surface lightness on the basis of Fe_2_O_3_ in the dry mix content. However, the development of a comprehensive prediction model needs additional experimental data.

**Fig. 18:**
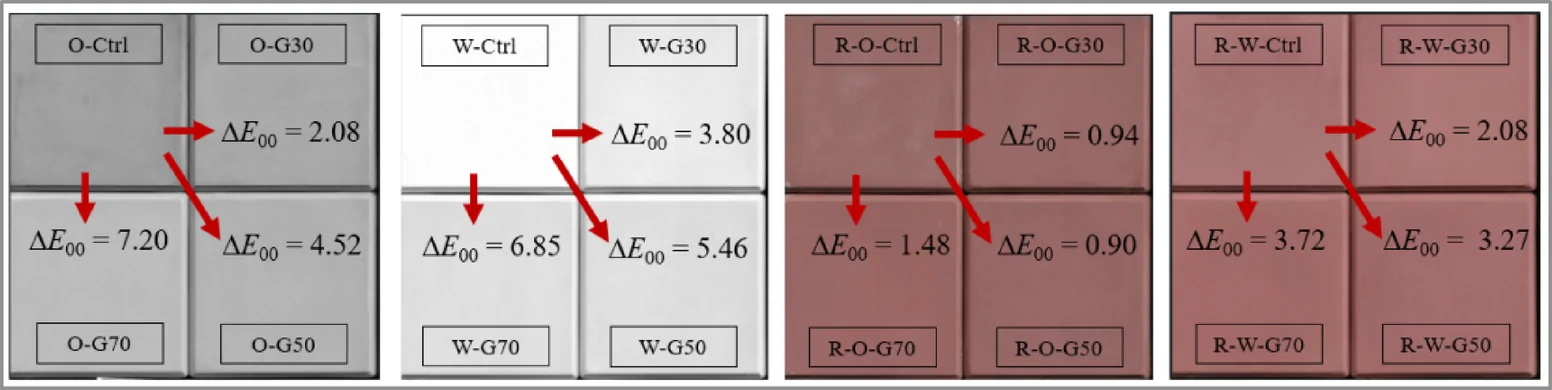
Colour difference (∆*E*_00_) on the surface of air-cured cement paste specimens for all mix series.

Figure 18 presents the scanned image and ∆*E*_00_ values of air-cured specimens for all four series mixes, where control mixes (mixes without GGBS) as the references for colour difference measurements. Increased in GGBS content increases ∆*E*_00_ value. The unpigmented specimens had higher ∆*E*_00_ value than the pigmented specimens. For example, when red pigment was added to the W-Ctrl and W-G70 mixtures, the colour difference decreased from 6.85 to 3.72. OPC-based red mixtures showed the lowest ∆*E*_00_ value (less than 1.5) regardless of the GGBS replacement rates. This result shows that the influence of binder on the pigmented paste was insignificant. Therefore, low-carbon white and grey Portland cement binders are suitable alternatives for pigmented cement binders.

### Colour difference due to different curing methods

**Fig. 19:**
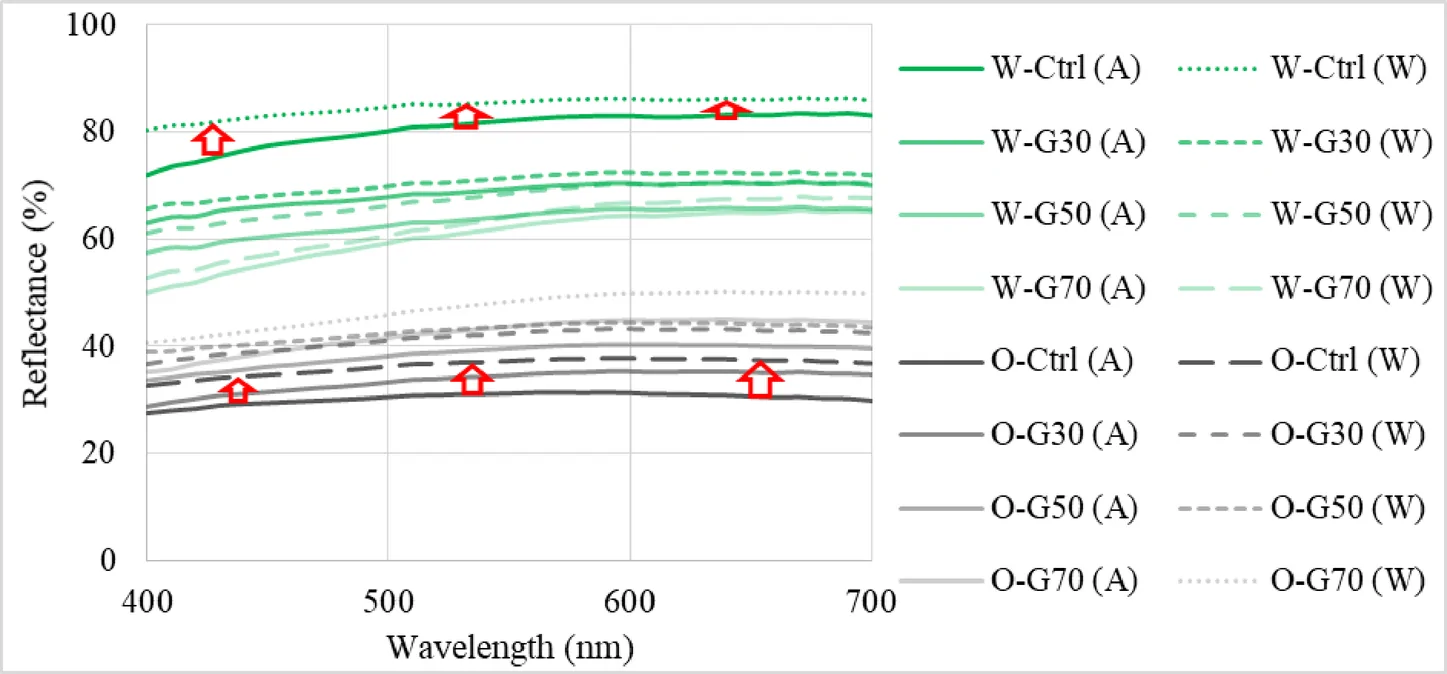
Surface spectral reflectance of air-cured (A) and water-cured (W) hardened cement pastes on day 29.

Figure 19 shows the spectral reflectance curves for unpigmented mixes (OPC- and WPC-based mixtures) on day 29. The results show an upward shift in the water-cured specimens compared with air-cured specimens. The shift is greater for OPC-based mixtures than for WPC-based mixtures, demonstrating that water curing provides a suitable surface characteristic for light reflectance. The results align with those reported by Qin et al.^61^, who studied the solar reflectance (280–2500 nm) of OPC paste using GGBS as SCM. In their study, increasing GGBS replacement from 5% to 30% increased the solar reflectance from 0.25 to 0.27 for air-cured specimens. However, for moist-cured specimens, the solar reflectance increased to 0.34 and 0.38. They recommended moist curing for concrete products, such as paving blocks and roof tiles, because of higher albedo than their air-cured version and able to minimise the urban heat island phenomenon.

**Fig. 20:**
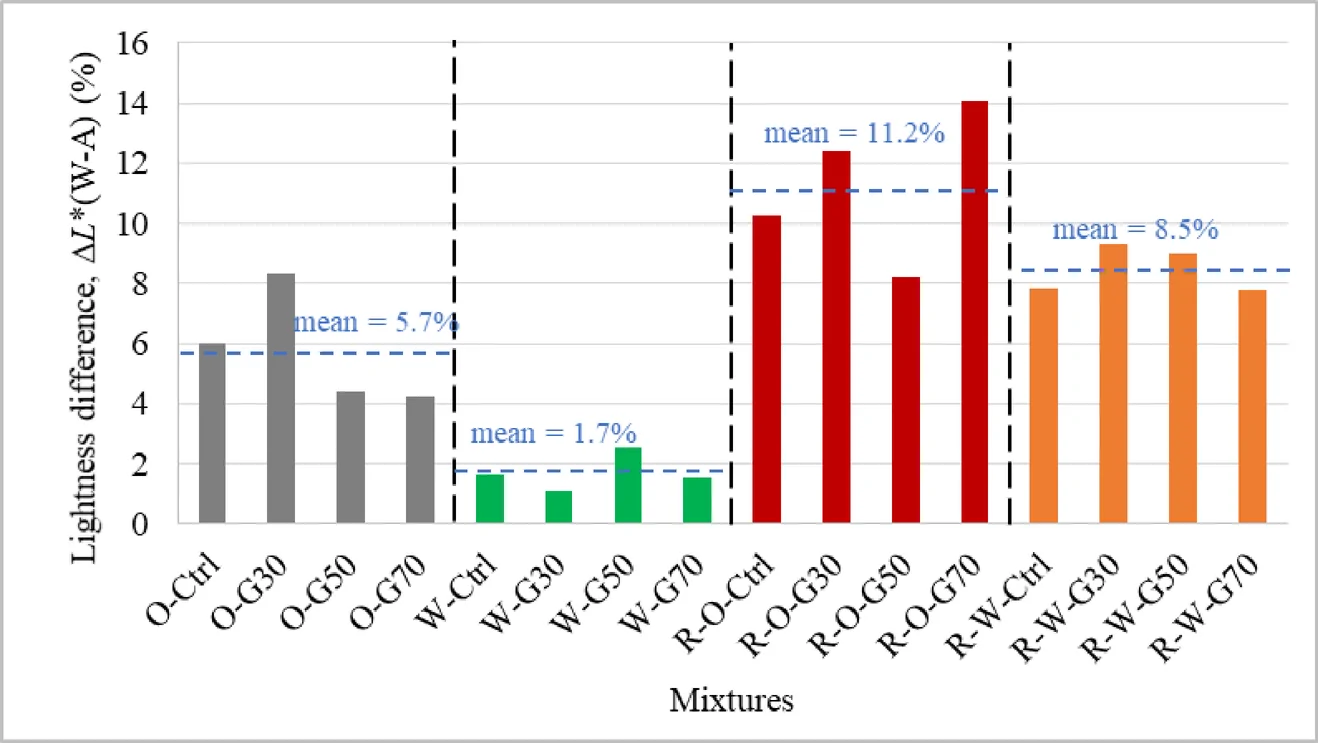
Lightness difference (∆L*) between water-cured (W) and air-cured (A) specimens for all mix series on day 29.

Figure 20 shows difference (in %) in L* between water- and air-cured samples, that is, ∆L*(W-A), for all mixes together with the mean value for each series. All the difference values were positive, showing the higher surface lightness of water-cured samples compared with air-cured ones. This observation is in agreement with the results of surface spectral reflectance (Fig. 18). In addition, the average value of each series (for pigmented and unpigmented groups) shows that OPC-based mixes have greater changes than the WPC-based mixes possibly because of the higher Fe_2_O_3_ and MnO content in the OPC (Table 1), which introduce large variations in light absorption.

### Limitations of the study

The colour of concrete is influenced by various factors, such as binder, filler, aggregate, pigment, water-cement ratio and surface texture. When concrete undergoes weathering and aging processes (e.g. carbonation, efflorescence, solar radiation, acid attack), the cement paste may decompose, leading to uneven surfaces and exposed aggregates. In this study, after demoulding and water curing, specimens were stored in the laboratory environment away from water, liquid, heat sources and ultraviolet radiation to avoid chemical reactions in the cement paste that could affect its surface appearance.

This study is a fundamental investigation that uses a spectrophotometer and the CIEDE2000 colour difference formula to measure and quantify the colour differences between different types of cement binders. This method can be used in colour analysis of other CBMs, provided that it has a flat surface that meets the spectrophotometer’s requirement.

Cement paste is the main component for all types of CBMs, such as cement mortar and concrete. Therefore, the results of this study can be generalized to other types of CBMs, although the results may not be entirely the same. Further research is needed for CBMs containing aggregates and other constituents. However, the result of this study can provide a reference while selecting binders for exposed concrete and for investigating the causes of colour differences in concrete or mortar due to binder factors.

## Conclusions

GGBS is a hydraulic-type supplementary cementitious material, with a high L* value and off-white colour. Owing to its excellent properties, it is used as partial replacement of cement in many major projects from low to high-volume. This study examines the influence of GGBS on the surface colour properties of hardened pastes prepared using grey OPC or WPC. The surface appearance of the specimens was studied visually and documented through scanned images with a flatbed scanner, and the colour was measured using a handheld spectrophotometer and evaluated using CIEDE2000 colour difference formula (∆*E*_00_). The following conclusions were drawn from the results of this study.

1. The colour uniformity of a hardened flat surface can be quantified using $\:{\varDelta\:E}_{00}^{im}$, in which *i* is the measured point and *m* is the mean point. This value should be reported together with the mean colour in a colour measurement report. The results showed that the water-cured WPC paste had the highest colour uniformity (∆*E*_00_ = 0.10 ± 0.03), whereas the air-cured OPC had the lowest (∆*E*_00_ = 1.29 ± 0.76).
2. Moisture on the surface of the cement paste led to a darker appearance; therefore, chromatic properties were measured only after the moisture on the surface and within the surface pores has completely dried.
3. Visual observation showed that the colour difference on the GGBS content of the pigmented specimens were generally lower than the observed in the unpigmented specimens. This observation was confirmed quantitatively using ∆*E*_00_.
4. The surface of the water-cured specimens was lighter in colour than air-cured specimens.
5. Overall, the results proved that incorporation of GGBS as a replacement for OPC or WPC, even at high-volumes, can produce CBMs without compromising its surface colour.

## Data Availability

Data will be made available on reasonable request.
